# HIV Infection: Time from Diagnosis to Initiation of Antiretroviral Therapy in Portugal, a Multicentric Study

**DOI:** 10.3390/healthcare9070797

**Published:** 2021-06-25

**Authors:** Vanessa Nicolau, Rui Cortes, Maria Lopes, Ana Virgolino, Osvaldo Santos, António Martins, Nancy Faria, Ana Paula Reis, Catarina Santos, Fernando Maltez, Álvaro Ayres Pereira, Francisco Antunes

**Affiliations:** 1 Escola Nacional de Saúde Pública, Universidade Nova de Lisboa, Av. Padre Cruz, 1600-560 Lisboa, Portugal; 2 Lean Health Portugal, Campus da Faculdade de Ciências da Universidade de Lisboa, Campo Grande, 1749-016 Lisboa, Portugal; lean@leanhealth.education (R.C.); maria.lopes@leanhealth.education (M.L.); 3 Instituto de Saúde Ambiental, Faculdade de Medicina, Universidade de Lisboa, Av. Professor Egas Moniz, 1649-028 Lisboa, Portugal; avirgolino@medicina.ulisboa.pt (A.V.); osantos@medicina.ulisboa.pt (O.S.); fmaltez@chlc.min-saude.pt (F.M.); alvaro.pereira@chln.min-saude.pt (Á.A.P.); fantunes@medicina.ulisboa.pt (F.A.); 4 Laboratório Associado TERRA, Faculdade de Medicina, Universidade de Lisboa, Av. Professor Egas Moniz, 1649-028 Lisboa, Portugal; 5 Unbreakable Idea Research, 2550-426 Painho, Portugal; 6 Centro Hospitalar Universitário de São João, Alameda Prof. Hernâni Monteiro, 4200-319 Porto, Portugal; antonio_rm26@hotmail.com; 7 Serviço de Saúde da Região Autónoma da Madeira, Av. Luís de Camões 6180, 9000-177 Funchal, Portugal; nancy.faria@gmail.com (N.F.); anapaulareis@sapo.pt (A.P.R.); 8 Hospital de Cascais, Av. Brigadeiro Victor Novais Gonçalves, 2755-009 Alcabideche, Portugal; catarina.esteves.santos@hospitaldecascais.pt; 9 Centro Hospitalar de Lisboa Central, Hospital Curry Cabral, Rua da Beneficência, nº 8, 1069-166 Lisboa, Portugal; 10 Centro Hospitalar Universitário Lisboa Norte, Hospital de Santa Maria, Av. Professor Egas Moniz, 1649-035 Lisboa, Portugal

**Keywords:** persons living with HIV, care continuum, ART initiation, systems for referral, care cascade

## Abstract

The benefits of antiretroviral therapy (ART) for persons living with HIV (PLWH) are well established. Rapid ART initiation can lead to improved clinical outcomes. Portugal has one of the highest rates of new HIV diagnoses in the European Union, and an average time until ART initiation above the recommendations established by the national guideline according to data from the first two years after its implementation in 2015, with no more recent data available after that. This study aimed to evaluate time from the first hospital appointment until ART initiation among newly diagnosed HIV patients in Portugal between 2017 and 2018, to investigate differences between hospitals, and to understand the experience of patient associations in supporting the navigation of PLWH throughout referral and linkage to the therapeutic process. To answer to these objectives, a twofold design was followed: a quantitative approach, with an analysis of records from five Portuguese hospitals, and a qualitative approach, with individual interviews with three representatives of patient associations. Overall, 847 and 840 PLWH initiated ART in 2017 and in 2018, respectively, 21 days (median of the two years) after the first appointment, with nearly half coming outside the mainstream service for hospital referral, and with observed differences between hospitals. In 2017–2018, only 38.0% of PLWH initiated ART in less than 14 days after the first hospital appointment. From the interviews, barriers of administrative and psychosocial nature were identified that may hinder access to ART. Patient associations work to offer a tailored support to patients’ navigation within the health system, which can help to reduce or overcome those potential barriers. Indicators related to time until ART initiation can be used to monitor and improve access to specialized care of PLWH.

## 1. Introduction

Since 1997, clinical efficacy and safety of a three-drug regimen, the highly active antiretroviral therapy (HAART), have been demonstrated to be effective in reducing the progression of HIV disease, as well as in decreasing AIDS incidence and mortality [1,2,3]. For persons living with HIV (PLWH), the benefit of antiretroviral therapy (ART) was determined by a large cohort study [4]. Results from this study, which were later supported by two large randomized clinical trials [4,5,6], showed a reduction in mortality among those who received ART when TCD4^+^ count was above 500 cells/µL, compared with those who started the treatment with values bellow this threshold [4]. Moreover, patients without other significant comorbidities and treated before serious immunosuppression can have a life expectancy similar to that of the general population [7,8]. Treatment can also prevent HIV transmission [9,10], with inevitable impact for public health.

Typically, ART begins weeks after HIV diagnosis, due to the fact that the cascade of HIV clinical care entails several stages from diagnosis to linkage to care, until ART initiation, which can present important barriers for treatment [11,12]. Notwithstanding, rapid ART initiation has proven to lead to better clinical outcomes (e.g., increased virologic suppression and retention in care, improved quality of life, AIDS- and non-AIDS-related morbidity and mortality prevention) and to important benefits in settings where there are long delays due to extensive patient preparation prior to ART initiation [13,14,15]. Moreover, treatment for PLWH can be initiated on the day of the diagnosis without any impact on ART safety or acceptability [13]. Same-day ART initiation may shorten the time to virologic suppression, improving retention in care with virologic suppression among patients with early clinical HIV diseases [13,14,16]. Indeed, the rapid initiation of ART, including same-day ART, is a World Health Organization recommendation [17,18].

Recent evidence suggests that there has been a dramatic and continuous decline in time from diagnosis to ART initiation, with significant benefits for treatment and prevention at the community level [19]. Studies examining changes in time to ART initiation over time, and factors contributing to its evolution, reinforce its valuable use as informative indicators since they have proven to be sensitive to changes in treatment patterns, accounting for both recently diagnosed and existing untreated patients. In addition, they can be made immediately or rapidly available. When focusing on the evaluation of quality of care and equity in access to care at the institutional level, within and between care organizations, under an approach and culture of continuous progression and improvement cycles, the current metrics of the HIV care cascade 90-90-90 (set up as an United Nations (UN) goal for HIV treatment) can fall short [19]. Time to ART can be used to better characterize the coverage of clinical care and guide the implementation of interventions at the different stages of the cascade to improve existing interventions. More specifically, HIV is a chronic manageable disease, much dependable on several individual risk-behaviors. Therefore, a better understanding of the indicators contributing to the forward and backward movements in disease care and ART initiation, namely perception of disease susceptibility and severity at short or long term, and/or of existing facilitators and barriers (cf. expanded health belief model (EHBM) [20]), has the potential to inform the development of targeted actions to vulnerable populations and key stakeholders.

In Portugal, we have one of the highest rates of new diagnoses of HIV infection and AIDS incidence in the European Union (against the background of the UN 90-90-90 HIV treatment target for 2020 [21]). A national guideline was established in 2015, determining how (and when) the referral process after HIV diagnosis should take place. Such a recommendation states that the first hospital appointment should take place within a maximum of 7 days after the request [22], although the maximum time between the laboratory confirmation of the diagnosis and the ART initiation is not defined by the guideline. With the publication of this guideline, monitoring indicators for people diagnosed with HIV and AIDS, under treatment and with controlled infection, had to be defined to assure a follow-up of the promoted programs for prevention and screening, as well as for early diagnosis and referencing accessibility to treatment by the health authorities. In the first two years after the implementation of this guideline, the average time from diagnosis to ART initiation corresponded to 68 days [23], which was largely above the evidence that points to the benefits of a rapid ART initiation, namely within 14 days after the diagnosis [24,25,26].

This study aimed to determine the time from the first hospital appointment after HIV diagnosis to ART initiation in Portugal, in the 2-year period of 2017–2018. Specific objectives included: (i) to characterize the population receiving ART and the origin of referral according to the national guideline; (ii) to compare the time to ART initiation (namely, time from the first specialized hospital appointment to ART prescription, and from prescription to collecting ART from hospital pharmacy) and the number of patients in treatment between different Portuguese referral hospitals for HIV treatment; (iii) to understand, from the perspective of different patient associations, the journey of the person living with HIV in Portugal, from the moment of hospital referral to ART initiation.

## 2. Materials and Methods

To answer the objectives of the study, two approaches were followed: a quantitative one, with analysis of hospital records, and a qualitative approach, which included three individual interviews with representatives of three patient associations.

### 2.1. Quantitative Approach

The quantitative approach aimed to characterize the patient population of newly HIV-diagnosed patients (i.e., with the date of HIV diagnosis in 2017 or 2018) initiating ART and the time elapsed from the first hospital appointment to ART initiation, in the main Portuguese referral hospitals for HIV treatment.

#### 2.1.1. Participants

In all adults living with HIV, ART should be initiated regardless of clinical stage and at any CD4 cell count [27]. In Portugal, HIV-related health appointments as well as the ART are fully covered by the national health service, assuring the universal (and free) access of patients to healthcare. The Portuguese Directorate General of Health established guidelines on how the referral process to access treatment should take place, both for people newly diagnosed with HIV, or with a HIV reactive test waiting for laboratory confirmation, coming from services of the National Health Service (NHS) or from other entities that settled agreements for the provision of health services [22]. Accordingly, hospital referral should be linked to the on-time appointment, an integrated system based on a national electronic referral system for requesting first specialist appointments in the Portuguese NHS, which allows greater efficiency and management of processes and resources [28]. In addition to this on-time appointment route of referral, PLWH can also be referred informally by hospital services or specialties, or by community-based screening institutions (e.g., non-governmental organizations [NGOs]).

As referred above, the guidelines determine that for cases of HIV infection or of HIV reactive test, the first hospital appointment should take place within a maximum of 7 days, counting from the registration date in the informatic system of the request [22]. The referral should be done to one of the NHS institutions that are integrated in the National Hospital Referral Network for HIV Infection [29,30]. Despite the existing evidence regarding the benefits of a rapid ART initiation (e.g., within 14 days after the diagnosis) [24,25,26], the maximum time between the laboratory confirmation of the diagnosis and the ART initiation is not defined by the Portuguese guidelines.

In this study, all individuals who had their first appointment date between 1 January 2017, to 31 December 2018 in one of the participating hospitals of the study were included.

#### 2.1.2. Study Procedures

Six Portuguese hospitals belonging to the national referral network for HIV infection were invited to take part in the study (non-probabilistic sample). These hospitals were selected taking into account the following criteria: (i) national geographic distribution (mainland Portugal and Autonomous (Islands) Regions), (ii) coverage of the highest number of newly HIV cases per year (high caseload; overall, invited hospitals follow around 80% of new HIV diagnostics per year), and (iii) sociodemographic heterogeneity of the followed HIV patients (i.e., migrant vs. non-migrant population, living in an urban area vs. living in non-urban region) and type of hospital (public vs. public–private partnership management model). After reaching out firsthand every HIV department director, by telephone or email, the contact was followed by a face-to-face meeting to discuss and refine the details of the study.

After confirming willingness to participate in the study, formal authorization was requested from the ethics committee of each hospital. Following the approval of each ethics committee, a structured data collection form was prepared and sent by email to each department director between February and October 2020. An anonymized database was received by one designated member of the research team, via email, from each of the five hospitals participating in the study. One of the enrolled hospitals was not able to collect the data in time for the analysis and was not included in the study.

#### 2.1.3. Variables/Indicators

Data records of patients fulfilling the inclusion criteria were date of birth, gender, origin of referral (on-time appointment or not), date of the first hospital appointment, date of ART prescription, and date of ART collection.

#### 2.1.4. Statistical Analysis

The received databases were uniformized and data cleaning procedures were conducted before the analysis: (i) elimination of duplicates; (ii) elimination of entries not following the inclusion criteria (e.g., first hospital appointment before 2017 and after 2018); (iii) elimination of entries with a previous HIV diagnosis; (iv) codification of potentially identifiable variables, including the attribution of unique identification codes to each entry; (v) use of the same nomenclature for the same information (e.g., NA for not available data).

Data analysis was carried out using SPSS Statistics for Windows, version 26.0. Descriptive statistics were used to summarize continuous variables using central tendency measures (means and/or medians, and percentiles 25 and 75) and respective dispersion measures (standard deviation or interquartile range). For categorical variables, the absolute number of individuals per category and respective percentages were determined.

Feedback from the data analysis was sent back to the participating hospitals to ensure cross-validation.

### 2.2. Qualitative Approach

A qualitative approach was applied as a sequential phase of the quantitative approach to provide additional understanding and insights that cannot be collected through the quantitative approach to explore and define the journey of the PLWH, from the moment of hospital referral to ART initiation, focusing the identification of main barriers to healthcare navigation and possible solutions to overcome or minimize the impact of those barriers. As a result of the rate of referrals identified as non-on-time appointments and the experience and specialized knowledge recognized in the work and practice of patient associations in Portugal in the field of HIV, an interview approach was designed to collect and gain an understanding of their experience about the current practices and processes of care, navigating and supporting PLWH. Three exploratory individual interviews with representatives of three patient associations were conducted between 12 October 2020 to 4 November 2020.

#### 2.2.1. Sampling Strategy and Participants’ Characteristics

The selection of the interviewees followed a purposive sampling approach to guarantee the diversity of the served populations according to geographic distribution of the hospitals enrolled in the study and a settled relationship of collaborative referral between organizations, hospitals, and community-based institutions. Respondents were recruited through mailed letters of invitation to each president (or similar leader position) of the three identified patient associations, asking for key actors with experience in developing pathways between community-based services and hospital care, supporting the navigation of people with an HIV reactive test throughout referral and linkage to care, and hospitals’ care services for PLWH. A respondent from each association was identified and all volunteered to take part in the study.

Due to the level of expertise and experience of the interviewees about the subject of the study, a satisfying level of data saturation was achieved with the three in-depth interviews regarding main barriers and facilitators that may hinder or decrease time to ART.

#### 2.2.2. Context, Methods, and Instruments of Data Collection

The interviews were conducted in a mixed format, face-to-face and online, following a semi-structured topic guide (Table 1). Interview questions were framed by the HIV treatment initiation model [13], which offered a structure of key milestones of care between testing and ART uptake, and were designed to ameliorate the understanding of the pathway between community-based testing and ART initiation.

The face-to-face interview was conducted in a private room of the corresponding patient association. For online interviews, it was assured that only the interviewer and the interviewee were attending to it. The interviews were conducted by one of the researchers, a psychologist trained in this method of data collection, and had an average length of 61 min (range between 50 and 74 min).

#### 2.2.3. Data Analysis

The interviews were audio recorded and fully transcribed by the same researcher who had conducted them. A thematic analysis was made for the full corpus of the interviews. The inductive thematic [31] analysis followed an open coding process, organizing key phrases into topics of discussion and continued to identify sub-themes associated and illustrated by quotes, mainly focusing on similarities and differences between respondents and between hospitals practices.

Data were anonymized using letters and numbers as an interviewee code (PA1, PA2, and PA3), and individual identifiers were limited by only including the name of the patient association of the participants. Anonymized transcripts and data analysis were sent back to the participants for interpretation validity (no corrections were suggested), controlling the risk of researcher bias at the analysis phase.

### 2.3. Ethical Issues

The study was developed according to the Declaration of Helsinki principles [32]. Approval from the ethics committees of the five hospitals was received prior to the beginning of the study. The sources of databases received from the participating hospitals were anonymized, so no identifying personal data were accessed by the researchers.

Regarding the qualitative component, the researcher previously debriefed the interviewees regarding data collection, use, and storage, and collected their informed consent. Confidentiality was assured to the interviewees and an identification code for anonymization was attributed to each individual record. Data were stored on the computer of the researcher protected by password.

## 3. Results

### 3.1. Patients on ART in 2017 and 2018

Between 1 January 2017, and 31 December 2018, 1687 patients initiated ART.

Demographic characteristics of the patients are presented in Table 2. Records received from the different hospitals were not received as uniform as requested. As can be observed in the following table, data regarding gender and age were only available for Hospitals A, C, and E. Overall, more men (76.1%) were on ART, a difference observed in all the hospitals that provided data regarding gender. Most participants in the three hospitals who sent this information were 20 to 59 years old. Although several differences were observed between hospitals, most PLWH came from non-on-time appointments.

Nearly the same number of individuals diagnosed with HIV initiated ART in 2017 (847) and in 2018 (840). The same tendency was observed in an analysis by hospital (Table 3).

### 3.2. Time from the First Hospital Appointment to ART Initiation among Newly HIV Diagnosed

Overall, ART initiation occurred 21 days (median; mean: 60; 60 days) after the first appointment in the hospital setting, with a variation between hospitals being observed, ranging from 17 to 39 days. The percentage of patients who initiated ART less than 14 days after the first hospital appointment was 38.0% (Table 4). Differences were observed between hospitals regarding extreme cases: in hospital A, 7.6% of the patients took more than a year to collect ART after the first hospital consultation; for hospital B, this happened only for 1.8% of the cases; for hospital C, the time between the first consultation and ART prescription (used as a *proxy* for ART initiation) happened in 1.4% of the individuals; hospital D did not have any cases where the collection of ART took more than a year; and in hospital E, only one case was observed (data not reported in the table).

### 3.3. HIV Care Continuum: From a Community-Based Positive HIV Test to ART Initiation

Patient Associations’ experiences regarding referral procedures and time until treatment seem to vary between hospitals. Formal referral protocols with community-based screening institutions coexist with informal (gentlemen’s) agreements.

*“We have different kind of issues, first geographic disparities, everything changes depending on the geographical area.”*

(Patient association [PA] 2)

*“From community-based testing, referral (to HIV care) has always been done informally, it’s done by email and through physicians with whom we work closely, or via telephone for urgent cases. Now one of our units had begun testing the referral system used by primary health care units, called on time appointment.”*

(PA3)

*“We have a referral protocol, that with some hospitals is agreed informally, and we have a clinical point person, who immediately schedules the appointment.”*

(PA2)

*“We always follow the procedure of delivering the referral form by hand. But when it´s an urgent case we contact the department director via telephone.”*

(PA1)

Referrals were described as following preferential relationships with some hospitals, even though individual preference is always respected.

Patient Associations frequently act as case managers and mediators, offering tailored support targeting the navigation of the health system and the overall treatment process to reduce or overcome potential barriers that make access to care more difficult, namely regarding scheduling of appointments (and stress related to the waiting times), psychosocial and economic-financial support, articulation of resources and flexibility of responses, or multichannel communication.

*“We can follow up and support each person who test positive to HIV in our community services, through referral to the first appointment. we have workers who personally navigate individuals through the hospital setting for the first appointment (…) because our hospitals are not easy to navigate, especially for immigrants (…).”*

(PA3)

*“But the process of navigating facilities for the first medical hospital visit is not easy (…), and this is one of our goals, to guide people through hospital facilities in the beginning of their HIV care pathway (…) and fortunately, for most of them, we aren´t needed anymore.”*

(PA1)

*“As expected, the interim period of one month will cause suffering for some who are waiting for HIV confirmatory results. (…) They may come to believe they will be the exception, (the test proves they aren´t infected with HIV), one month post confirmatory results is too long (…). When needed, our professionals work to engage with and keep close contact with each person along that interim period, to help coping with anxiety.”*

(PA1)

The participants acknowledged this informal proximity between the associations’ mediators and hospitals’ focal point or reference clinicians as a key condition to better manage more complex and urgent cases.

*“Along the implementation of routine HIV testing services, one of our first tasks was to navigate each hospital facilities and routes as an anonymous user. (…) once done became obvious that previous experience was critical before sharing information on accessibility with patients, along with scheduling their first appointment.”*

(PA2)

*“It´s the physician with who we work closely that reports us missed first appointments, and we most often try to reach the person to offer a rescheduling. We look for the reasons for not attending and try to reschedule more accordingly to their availability. (…) Our role is to favor at most health care access and suit it best to each person’s life.”*

(PA3)

#### 3.3.1. Barriers to a Reduction in the Time between Referral and ART Initiation

The interviewees expressed concerns regarding people from key affected populations facing specific unmet needs that could be more challenging to adhere and link to care (e.g., immigrants, sex workers, people who use injectable and non-injectable drugs).

Several psychosocial, procedural, and administrative barriers were identified—balancing personal and professional life with organizational processes and administrative procedures was one example (e.g., low flexibility in clinical hours, long waits for physician appointments, undocumented immigrants). In addition, stigma was pointed out as hindering access to ART.

*“Barriers (…) are easily seen and of importance in the immigrant population.”*

(PA3)

*“In their hierarchy social issues become first than health (…).”*

(PA1)

*(…) Affected populations are different. If we focus on people facing economically deprived situations or facing cognitive impairment deficits, they ask us for support to navigate the system and first-visit escort or transport to the HIV care and treatment facility. But if we talk about primary infections, mainly involving men who have sex with men, who demonstrate better knowledge about the infection, they value more support via telephone, message exchange, social networks.”*

(PA2)

*“(…) We look for the reasons and try to understand, and sometimes is a matter of basic issues, as being unable to afford a bus ticket. To give you an example, a person can wait for more than an hour for the appointment, and some are unable to get that time off work. One of the persons, that we support, reported that he had to leave as he was risking being discharged from work. And for the physician he missed the appointment because he didn´t wait. We can bring the patient perspective to the physician and conciliate to reschedule and benefit from the tolerance between both sides.”*

(PA2)

#### 3.3.2. Good Practices to Reinforce Care Continuity between HIV Diagnosis and ART Initiation

The proximity effect of close interactions between community-based organizations and hospital caregivers (i.e., hospital care teams) seems to act as a driver for developing local solutions and tailored responses to unmet needs, creating an opportunity and space to compromise, negotiate, and test new solutions.

*“(…) And we started working together. I see that as the main reason that led us to accomplish these extraordinary results. The opening of doors in hospitals to work closely with patients’ associations offered an opportunity to listen to each other, brought awareness for some of the barriers faced, and how we could find solutions.”*

(PA2)

Several practices and experiences that can work to decrease the time to ART initiation were identified: (a) confirming a first appointment date at the time of the HIV testing; (b) having responsive scheduling that accommodates next-day appointments after testing; (c) having a focal point through which to mediate communication and more easily connect people to hospital care; (d) regularly sharing information and statistics between hospital HIV departments and community-based organizations; (e) close interaction between community-based workers and hospital caregivers as a means to develop trust relations and encourage testing new interventions focusing on the delivery of care and jointly endorsing research close to practice.

*“We try and invest our efforts to guarantee that we give an appointment date for a physician´s visit to each person testing reactive to HIV on the moment of their HIV test. Obviously that´s not a practice reachable with all hospitals. (…) To our knowledge that´s the best practice, even to avoid drop-outs, firming an appointment date after an HIV testing reactive, and assuring readiness to it, as a way of offering some comfort to that person.”*

(PA2)

*“Well, this is an overly sensitive matter, test and treat early, before having some of the lab test results, (…). When we began to offer a post confirmatory test in our community-based testing services, we proposed to one referenced hospital for HIV care, the possibility of having a responsive scheduling that accommodates next-day appointments, making early treatment happen. Because, in fact, this is a recommendation whether from the WHO or from the ECDC (…).”*

(PA3)

*We have a fast-track procedure (…), for suspected cases that are in the so-called acute phase, and we have managed to referral some, not as many as we would like. (…) This has been a reality for a small number of cases (…).”*

(PA3)

## 4. Discussion

Since the implementation of the national guidelines in 2015 in Portugal, which define how the referral process should take place for individuals newly diagnosed with HIV so they can access proper treatment [22], the time until ART initiation was only assessed for the first two years. This evaluation revealed that, for those patients, the time interval between the diagnosis and ART initiation was, on average, 76 days in 2015, 69 days in 2016, and 68 days in 2017, which is well above the recommendation [23]. In this study, the years of 2017 and 2018 were analyzed, with it being found that the mean time from the first hospital appointment (and not from HIV diagnosis, unlike what was reported in 2015–2017) until ART initiation was of 60.6 days (with a median of 21 days). Considering a threshold of 14 days after the diagnosis (taking into account the limit pointed out in other studies; e.g., [25,26,33]), we observed that, in total, only 40% of the patients initiated ART in less than 14 days, with considerable differences between the five hospitals. Despite the discussion that still persists around the optimal time for ART initiation (within days or weeks after the diagnosis), clinical benefits of early initiation are well established, namely the reduction of AIDS-related morbidity and mortality, and of the risk of HIV transmission due to behavior modification [3,5,9,10,34,35,36]. In HIV-infected people, ART reduces the viral load (RNA-HIV levels) in plasma, female genital secretions, semen, and also in secretions from the rectum. Given the association between viral load and the risk of transmission, and knowing that ART is not solely responsible for reducing the transmission of HIV infection, it can prevent sexual transmission of HIV by reducing reservoir infectivity [9,15].

Several countries are now monitoring the continuum of care in HIV in order to achieve better ART coverage [37]. However, challenges in comprehensively understanding the barriers to initiation of ART can hamper rapid ART initiation [19,38]. For example, complete population-based data from all jurisdictions have been deemed as necessary to properly assess changes in the continuum of care over time [19]. In this study, an effort was made to have a geographical distribution of the analyzed data. Our findings have demonstrated large inter-hospital differences from the first specialist appointment after HIV diagnosis until ART initiation, ranging from 17 to 39 days (median). All hospitals performed above the recommended interim period. These results can be explained by the practice of deferring ART initiation until having the results of laboratory tests, such as drug-resistant HIV testing. [23]. These data suggest that more efforts should be made in the future to have a more complete view of the continuum for HIV care, namely to have a longitudinal representation of patients diagnosed with HIV, to collect disaggregated data to better monitor the time spent at each point of the continuum, and to gather more complete data from each hospital of the Portuguese Network for HIV Infection [19].

The experience and practice of navigation between a community-based positive HIV test and ART initiation, established from the interviews, allowed the identification of psychosocial, procedural, and administrative barriers for decreasing time to ART initiation following an HIV diagnosis. At the hospital level, low flexibility in scheduling appointments to suit working times, long waiting times for appointments, and the burden and bureaucracy of administrative procedures were some of the examples of factors hindering the access to proper HIV treatment. Affected key populations were also signaled as facing more challenges in linking and adhering to care (e.g., immigrants, sex workers, people who use drugs), relating to financial difficulties to afford transportation, low health literacy, stigma and discrimination, and competing legal demands. These barriers are in line with the literature, which has been reporting different types of barriers to ART access and adherence, namely related to the burden of medication and health concerns, stigma and discrimination, conflicting family responsibilities, and low flexibility for scheduling routine visits [39,40]. In fact, our findings allowed us to shed light on some of the factors that potentially can contribute to the increase of risk behaviors for HIV. According to the EHBM [20], if a negative health condition (e.g., HIV) is perceived as avoidable, the individual will not only engage in health-related actions that will protect him/her from the disease, but will also believe the he/she can successfully do it. In this context, it is extremely relevant to eliminate or minimize obstacles for treatment. Waiting time for starting the treatment constitutes a main accessibility-related obstacle. A better understanding of the factors happening to influence the continuum of the HIV care process can therefore inform the development of intervention programs targeting vulnerable populations and key stakeholders.

Some promising and good practices that were shared highlighted the importance of a proximity between community-based organizations, health professionals, and persons living with HIV. Some of the promising practices (e.g., first consultation at the same time as, or immediately after, the reactive test; existence of a mediator between the patient and the hospital; regular sharing of information between referral hospitals and the community-based organizations) are in line with what has been reported in the literature as facilitators to ART adherence, such as family/social support, good relationship with the health provider, or perceived health benefits [39,41,42,43].

Although this is the first Portuguese study, with a good national coverage, some limitations should be discussed. First, not all hospitals from Portugal’s mainland and Autonomous Regions, were involved. Nevertheless, a good representation of the National Hospital Referral Network for HIV Infection was assured, with the sample covering nearly 80% of new HIV diagnoses. Second, although a structured form was sent to each of the participating hospitals, due to the burden of data collection, a consequence of different levels of deficiency of record-keeping and access to routine compilations of data on HIV cases, not all hospitals were able to fulfill the required data list and a substantial disparity was faced relating to data completeness, limiting the inferences and analysis that could be drawn. Extensive data cleaning procedures were applied to pursue some uniformization between databases. Even so, this highlights the need to implement regular procedures of data reporting as already implemented in other countries [37]. Indeed, other variables should be included in future studies to better understand the HIV care continuum, namely the time between the referral and the first hospital appointment, as all the stages have an impact on the overall care system and on the quality of life for those living with HIV. Moreover, in this study, the variable used to determine ART initiation was the lag time between the date of the first appointment until the date of collection of the prescription. However, previous existing data [23] used the date of the diagnosis and not the date of the first appointment, which makes it difficult to compare the evolving trends in the existing access to HIV care. On the other hand, this cannot be used to determine patient medication compliance (presumably when the beneficial clinical outcomes would begin), as the relevant data were not possible to obtain from the participating hospitals. Our purpose in this study was to assess HIV care processes and the time until ART initiation. So, future studies should address this gap between the moment of prescription and patients’ actual beginning and continuing of the treatment regimen. Also, the impact of rapid ART initiation on patient adherence to the therapeutics can be further assessed. With so many variables at stake, a more standardized availability of the data should be pursued so that a more reliable comparability can be established to assess the HIV care process. Finally, although the interviews in the qualitative component were planned to shed light on some of the missing information collected in the quantitative part, the exploratory nature of the study did not allow us to clarify potential differences (namely regarding barriers to HIV care) between hospitals, which would be an interesting topic to develop. In addition, the interviews took place only in 2020. Therefore, a recall bias can hinder some of the told stories. Even so, the sustainability and the referral model in HIV care did not change in between 2017 and 2018, which reinforces some of the conclusions drawn.

## 5. Conclusions

In Portugal, a systematic monitoring of the referral process until ART initiation is still lacking. This was a first effort to better understand how well different hospitals can comply with the national guidelines in terms of accessing care and treatment. Time to ART as an indicator is sensitive to changes and is a readily and immediately available indicator. As cascades may not reflect these changes, these metrics should be used to benchmark the center’s performance and establish indicators of best practice. Moreover, access to care and HIV treatment seems to be maximized when the psychosocial, economic, and administrative barriers confronted by key affected populations are overcome. Successful practices must be open to discussion between stakeholders in an accurate manner and under a continuous quality improvement approach.

## Figures and Tables

**Table 1:** Topics of the interview guide.

Dimension	Sub-Dimension
1. Referral and health system navigation	Detailing the experience and practice of navigation between a positive HIV test and ART initiation in Portugal. Identifying procedures variability and time intervals between care referral, linkage to care, and ART initiation
2. Access to referral services and access to care	Exploring the main facilitators, challenges, and barriers to accessing referral services, starting medical care, and ART initiation
3. Good practices	Collecting good practice examples at national and international levels with possible impacts on the path between referral, diagnosis, and ART initiation

**Table 2:** Demographic characteristics of patients on ART ^a^, by referral hospital ^b^, in 2017–2018.

	Total *n*	Hospital A *n* (%)	Hospital B *n* (%)	Hospital C *n* (%)	Hospital D *n* (%)	Hospital E *n* (%)
Gender			-		-	
Male	804	614 (77.2)		139 (77.7)		51 (61.4)
Female	253	181 (22.8)		40 (22.3)		32 (38.6)
Age ^c^			-		-	
<15 years	12	11 (1.4)		1 (0.6)		0 (0.0)
15–19 years	8	4 (0.5)		3 (1.7)		1 (1.2)
20–29 years	261	202 (25.4)		48 (26.8)		11 (13.3)
30–39 years	315	249 (31.3)		45 (25.1)		21 (25.3)
40–49 years	248	198 (24.9)		26 (14.5)		24 (28.9)
50–59 years	137	87 (10.9)		34 (19.0)		16 (19.3)
>59 years	76	44 (5.5)		22 (12.3)		10 (12.0)
Mean (± SD)	39 (±12.98)	38 (±12.41)	-	41 (±15.00)	-	44 (±12.29)
Minimum	1	1		1		19
Maximum	83	83		83		79
Origin of referral						
On-time appointment	287	121 (15.2)	67 (11.1)	92 (51.4)	0 (0.0)	7 (24.1)
Not on-time appointment ^d^	676	402 (50.6)	84 (14.0)	85 (47.5)	83 (100.0)	22 (75.9)
Not available	724	272 (34.2)	450 (74.9)	2 (1.1)	0 (0.0)	0 (0.0)

^a^ For hospitals A, B, D, and E, data refer to the date of ART collection; for hospital C, the date of ART collection was not provided by the hospital, with only the date of ART prescription being available. ^b^ Data regarding gender were unavailable and age for hospitals B and D. ^c^ Age at first hospital consultation. ^d^ Includes patients from primary health centers outside the on-time appointment, from other hospital services, or from NGOs.—No data available (i.e., not received from the hospital).

**Table 3:** Patients on ART ^a^, in 2017 and 2018, by referral hospital.

	Total *n* (%)	Hospital A *n* (%)	Hospital B *n* (%)	Hospital C *n* (%)	Hospital D *n* (%)	Hospital E *n* (%)
2017	847 (50.2)	410 (51.6)	290 (48.3)	92 (51.4)	42 (50.6)	13 (44.8)
2018	840 (49.8)	385 (48.4)	311 (51.7)	87 (48.6)	41 (49.4)	16 (55.2)

^a^ For hospitals A, B, D, and E, data refer to the date of ART collection; for hospital C, the date of ART collection was not provided by the hospital, with only the date of ART prescription being available.

**Table 4:** Time (days) from first hospital appointment to ART initiation, by referral hospital, 2017–2018.

	Total *N* = 1687	Hospital A *n* = 795	Hospital B *n* = 601	Hospital C ^a^ *n* = 179	Hospital D *n* = 83	Hospital E *n* = 29
Mean (95%CI)	60.60 (52.51–68.69)	83.96 (69.47–98.44)	35.45 (28.36–42.54)	71.06 (60.06–82.05	29.82 (20.12–39.53)	71.96 (36.02–107.91)
Quartile 25	0.00	0.00	0.00	0.00	14.00	28.00
Median	21.00	22.00	17.00	26.00	22.50	39.00
Quartile 75	40.00	49.00	34.00	46.25	30.00	82.50
IQR	40	49	34	46	16	55
% of HIV patients with ART initiation < 14 days	38.0% ^b^	36.6%	43.9%	5.7%	15.7%	17.2%

^a^ For hospitals A, B, D, and E, data refer to the date of ART collection; for hospital C, the date of ART collection was not provided by the hospital, with only the date of ART prescription being available. ^b^ Due to missing data for Hospital C, the total does not include this hospital.

## Data Availability

Publicly available datasets were analyzed in this study. These data can be found here: https://leanhealth-pt.maps.arcgis.com/apps/Cascade/index.html?appid=3215a89f3edf41629139e0191c9e093a (accessed on 10 June 2021).
